# *Leishmania guyanensis* M4147 as a new LRV1-bearing model parasite: Phosphatidate phosphatase 2-like protein controls cell cycle progression and intracellular lipid content

**DOI:** 10.1371/journal.pntd.0010510

**Published:** 2022-06-24

**Authors:** Alexandra Zakharova, Amanda T. S. Albanaz, Fred R. Opperdoes, Ingrid Škodová-Sveráková, Diana Zagirova, Andreu Saura, Lˇubomíra Chmelová, Evgeny S. Gerasimov, Tereza Leštinová, Tomáš Bečvář, Jovana Sádlová, Petr Volf, Julius Lukeš, Anton Horváth, Anzhelika Butenko, Vyacheslav Yurchenko

**Affiliations:** 1 Life Science Research Centre, Faculty of Science, University of Ostrava, Ostrava, Czech Republic; 2 De Duve Institute, Université Catholique de Louvain, Brussels, Belgium; 3 Faculty of Natural Sciences, Comenius University, Bratislava, Slovakia; 4 Institute of Parasitology, Biology Centre, Czech Academy of Sciences, České Budějovice (Budweis), Czech Republic; 5 Department of Parasitology, Faculty of Science, Charles University, Prague, Czech Republic; 6 Faculty of Science, University of South Bohemia, České Budějovice (Budweis), Czech Republic; Bernhard Nocht Institute for Tropical Medicine, Hamburg, Germany, GERMANY

## Abstract

Leishmaniasis is a parasitic vector-borne disease caused by the protistan flagellates of the genus *Leishmania*. *Leishmania (Viannia) guyanensis* is one of the most common causative agents of the American tegumentary leishmaniasis. It has previously been shown that *L*. *guyanensis* strains that carry the endosymbiotic *Leishmania RNA virus 1* (LRV1) cause more severe form of the disease in a mouse model than those that do not. The presence of the virus was implicated into the parasite’s replication and spreading. In this respect, studying the molecular mechanisms of cellular control of viral infection is of great medical importance. Here, we report ~30.5 Mb high-quality genome assembly of the LRV1-positive *L*. *guyanensis* M4147. This strain was turned into a model by establishing the CRISPR-Cas9 system and ablating the gene encoding phosphatidate phosphatase 2-like (PAP2L) protein. The orthologue of this gene is conspicuously absent from the genome of an unusual member of the family Trypanosomatidae, *Vickermania ingenoplastis*, a species with mostly bi-flagellated cells. Our analysis of the PAP2L-null *L*. *guyanensis* showed an increase in the number of cells strikingly resembling the bi-flagellated *V*. *ingenoplastis*, likely as a result of the disruption of the cell cycle, significant accumulation of phosphatidic acid, and increased virulence compared to the wild type cells.

## Author summary

Worldwide, over one million people are getting infected by the parasitic flagellates of the genus *Leishmania* annually leading to ~30,000 deaths. Notably, there is still no approved vaccine against human leishmaniases. A range of methods of forward and reverse genetics has recently been developed for several model *Leishmania* species. Unfortunately, these methods are often not transferrable to non-model species, which may be of even greater medical importance. *Leishmania guyanensis* is one of such cases. It frequently carries a symbiotic RNA virus that contributes to the development of a more aggressive form of leishmaniasis in an experimental murine model. In order to establish and optimize the system for genetic manipulations in *L*. *guyanensis*, we sequenced and annotated its genome. Next, we applied the CRISPR-Cas9 technology to target a gene of interest. This approach was validated by ablating a gene encoding a protein involved in lipid metabolism. In this work, we document that deletion of this gene leads to the disturbance of cell cycle and affects the ratio of critical intracellular lipids. We believe that our study will facilitate research into more effective treatment of leishmaniases.

## Introduction

Members of the family Trypanosomatidae (Euglenozoa: Kinetoplastea) are obligate parasites of insects, leeches, vertebrates, and plants [1,2]. In their life cycle, they have either one host (in overwhelming majority of cases an insect) or alter between two hosts in case of monoxenous and dixenous species, respectively [3,4]. Many dixenous trypanosomatids are medically and/or economically important [5–7]. Flagellates of the genus *Leishmania* infect reptiles or mammals causing a spectrum of diseases collectively named leishmaniases [8]. Over 350 million people, primarily in the tropical and subtropical regions, are at risk of infection [9]. These parasites are transmitted by bloodsucking phlebotomine sand flies (Psychodidae) [10] or biting midges (Ceratopogonidae) [11] and cause clinical symptoms ranging from mild skin lesions to fatal visceral organ failures.

*Leishmania guyanensis* is a causative agent of American tegumentary leishmaniasis (ATL). This chronic, latent and often metastatic disease manifests itself as single or disseminated cutaneous and/or mucosal lesion(s) (so called mucocutaneous leishmaniasis), followed by propagation of the parasite through the blood and lymphatic systems [12]. First described in Central America in 1954 [13], this *Leishmania* sp. can parasitize numerous mammalian species [14–16]. The molecular mechanisms, by which some ATL patients develop a more severe form of the disease, remain under-investigated. Notably, some isolates of *L*. *guyanensis* possess endosymbiotic *Leishmania RNA virus 1* (LRV1) of the family *Totiviridae* [17,18]. The presence of this virus has been linked to the increased disease pathology and elevated parasite load in animal models [19–22], as well as to the severity of human leishmaniasis and associated treatment failures, although the direct connection to the human diseases is still being debated [23,24]. The genomes of several *L*. *guyanensis* isolates have been sequenced, including isolates LgCL085 [25], 204–365 [26], and M4147 [27], with the former two draft-assembled.

The number of *Leishmania* species with sequenced and annotated genomes that are amenable to genetic manipulations and, thus, can be considered as models, remains limited [28]. Moreover, to the best of our knowledge, this list does not include any LRV-containing isolate, precluding detailed molecular studies into the undoubtedly important role viruses play in *Leishmania* biology and leishmaniasis etiology. The majority of currently used gene modification systems in trypanosomatids rely on variations of the CRISPR-Cas9 technology [29–32], although homologous recombination and other approaches remain in use [33]. The former approach is standardized, easy to scale-up in target screening, and allows analysis of multigene families.

In the current study, we have assembled and annotated the genome of LRV1-positive *L*. *guyanensis* M4147 and, by applying the CRISPR-Cas9 system, turned it into a model species by ablating a gene encoding phosphatidate phosphatase 2-like protein (*Lg*PAP2L). An ortholog of this gene is conspicuously absent from the genome of *Vickermania ingenoplastis* [34,35] making it an interesting target for further investigations. In general, phosphatidic acid phosphatases (EC 3.1.3.4, PAP) are divided into two types. The PAP1 enzymes are substrate-specific for phosphatidic acid (PA), Mg^2+^-dependent, N-ethylmaleimide-sensitive and soluble. They convert PA into 1,2-diacylglycerol (DAG) that can be subsequently used in the Kennedy pathway of glycerophospholipid biosynthesis [36]. In contrast, Mg^2+^-independent and N-ethylmaleimide-resistant PAP2 enzymes dephosphorylate a wide range of substrates, such as phosphorylated carbohydrates, peptides and lipids. They are involved in many cellular processes, such as vesicular trafficking, secretion and endocytosis, protein glycosylation, energy storage, and stress response [37–40]. A typical catalytic domain of PAP2 contains several short motives–KX_6_RP, PSGH, and SRX_5_HX_3_D (residues involved in catalysis are underlined) [36]. Lipid phosphate phosphatases (LPPs), a subgroup of the PAP2 superfamily unites membrane-integrated enzymes with their active site facing the outer environment [41,42]. Their typical substrates are lipid monoesters, such as lysophosphatidic acid, phosphatidic acid, sphingosine-1-phosphate (S1P), ceramide-1-phosphate (C1P), or diacylglycerol pyrophosphate (DAGPP) [43–45]. These enzymes are thought to regulate the level of intracellular bioactive lipid phosphates, which mediate intracellular signalling *via* a number of central and conserved pathways, including ERKs, mTORC1, Sos, Raf, phospholipase C-γ, sphingosine kinase-1, and NADPH oxidase [36]. A mammalian LPP2 was also shown to regulate cell cycle, although the molecular mechanism of its action was not elucidated [46]. Despite their importance, the PAP2 family proteins in protists remain understudied, with reports being restricted to just a few prominent parasites. In *Plasmodium falciparum*, the inhibitors of PAP2 proteins disrupt PA homeostasis and influence cell cycle progression [47]. Among trypanosomatids, PAP2 was studied only in *Trypanosoma cruzi*, where the decrease in its activity resulted in a concomitant increase in the PA content as the cells progressed from the epimastigote to trypomastigote stages [48]. Notably, other members of the PAP superfamily are present in the *V*. *ingenoplastis* genome (S1 Table).

## Results

### *Leishmania guyanensis* M4147 genome analysis

Following the mis-assembly correction, gap filling, and scaffolding, we obtained *L*. *guyanensis* MHOM/BR/75/M4147 genome assembly with the length of ~30.47 Mb and N_50_ value of approximately 1 Mb. It contains 35 chromosome-length scaffolds, 6 unplaced and 100 kDNA sequence contigs (S3 Table). In addition to a relatively high contiguity, the assembly almost lacks gaps and errors (the percentage of gaps is ~0.01%), as suggested by the high proportion of genomic reads mapping back to the assembly (~98%) and low content of homozygous single nucleotide polymorphisms (SNPs) (635 or 0.002%). The genome assembly of *L*. *guyanensis* MHOM/BR/75/M4147 is shorter than that of other representatives of the *Leishmania* (*Viannia*) subgenus analyzed here (S3 Table). However, the percentage of missing BUSCOs is the smallest (35.3%) among all the compared assemblies, including the “gold standard” *Leishmania major* Friedlin (36.8%). The genome annotation contains 8,273 protein-coding and 79 tRNA genes. The number of predicted proteins is lower than that in the larger genomes of *L*. *braziliensis* MHOM/BR/75/M2904_2019 (8,484) and *L*. *major* Friedlin (8,424), but higher than that of *L*. *panamensis* MHOM/PA/94/PSC-1 (7,748) with comparable genome size. Current assembly demonstrates high levels of gene order conservation compared to the assemblies of several other representatives of the *L*. (*Viannia*) group (S2 Fig).

### Predicted metabolism of *L*. *guyanensis*

The metabolism of *L*. *guyanensis* is very similar to that of its model relative *L*. *major* [49]. Our data are summarized in the S1 File and S4 Table. [50][51][52][53][54][55–57][58–60][49,61][62][63][63][64][49]

### Reconstruction of *Leishmaniavirus 1* sequence from total transcriptomic reads

We detected the presence of complete LRV1 genome in all wild type (WT) replicates of *L*. *guyanensis*, which can be easily assembled *de novo* with Trinity. The contig size and the average coverage were 5,265 nt and 2,277x, respectively. Best BLAST hit in the NCBI nt database was *Leishmania RNA virus 1* strain LRV1-LgyM4147 (KX808487), which had 100% query coverage and sequence identity of 99%.

### Establishment of CRISP-Cas9 system in *L*. *guyanensis*

To establish a new model species, we first introduced the episomal CRISPR-Cas9-dependent system for genetic manipulations [65,66] into *L*. *guyanensis*. Successful expression of *Cas9* was confirmed by RT-qPCR (S3A Fig) and Western blotting with anti-FLAG antibodies (S3B Fig). Since the expression of *Cas9* varied significantly between the drug-selected populations and clones, for all subsequent experiments we choose a clone with the highest expression (clone 5 in S3 Fig). Notably, the expression of *Cas9* does not affect cell division (S3C Fig).

### Ablation of LgPAP2L in *L*. *guyanensis* and establishment of the add-back line

For the proof of principle, we have chosen a gene *Lg_M4147_000239500* encoding a putative PAP2-like protein of the LPP subgroup. It will be referred to as *pap2l* hereafter. This choice was primarily determined by the fact that the ortholog of this gene is absent from the genome of *Vickermania ingenoplastis* [34,67], a member of rather unique group of trypanosomatids that uses two joined flagella to resist midgut peristaltic flow within the fly host [35].

**Fig 1:**
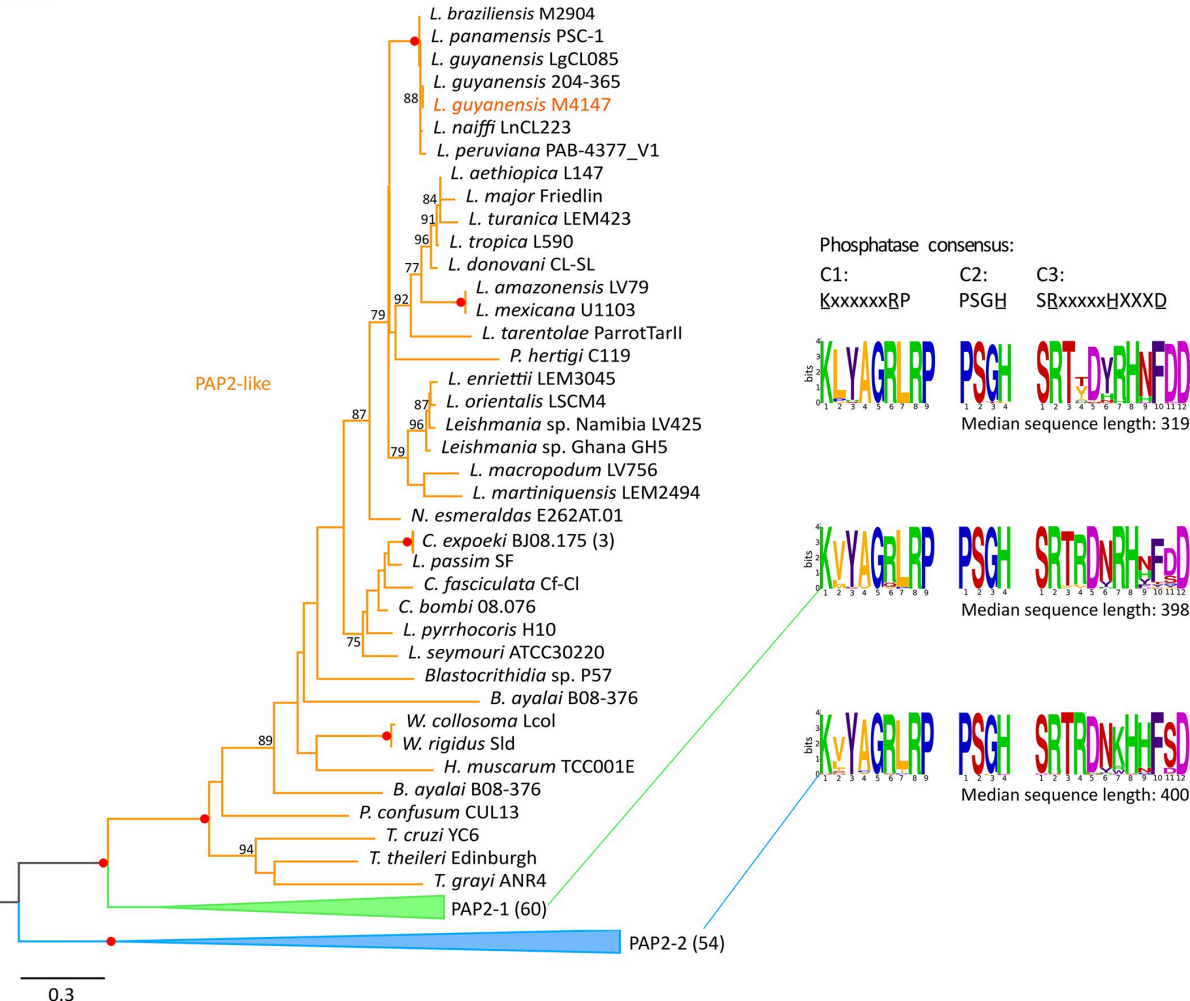
Maximum-likelihood phylogenetic tree of 155 PAP2 and PAP2L sequences of 50 kinetoplastid species. Only bootstrap supports over 75% are shown (red dots indicate maximal supports). The analyzed PAP2L of *L*. *guyanensis* is colored in orange. The tree is divided into three main clades colored in orange (clade containing the PAP2L investigated here and *L*. *major* Friedlin *LmjF*.*18*.*0430*), green (clade containing *LmjF*.*18*.*0440* and other 59 PAP2-related sequences, labeled PAP2-1), and blue (clade containing *LmjF*.*19*.*1350* and other 53 PAP-related sequences, labeled PAP2-2). Numbers of sequences within collapsed clades are shown in brackets. Weblogos of the PAP2 superfamily (PF01569) phosphatase domain are shown on the right for the PAP2L (top), PAP2-1 (middle), and PAP2-2 (bottom) groups. Median sequence length for the three major clades is also shown. Residues of amino acids are colored in accordance with their chemical properties: positive charged side chains are in green; negative charged side chains are in pink; polar uncharged side chains are in red; non-polar hydrophobic side chains are in orange; aromatic hydrophobic side chains are in purple; and special cases are in blue. Residues involved in catalysis are underlined. See S1 Table for additional information.

The maximum-likelihood phylogenetic tree of trypanosomatid PAP2 includes 155 sequences clustering into 3 major clades (Fig 1 and S1 Table), which we named PAP2-1, PAP2-2, and PAP2L for convenience. The phosphatase domain of the representatives of PAP2-1 and PAP2-2 is more closely related to the canonical PAP2 domain (PF01569 in the Pfam database), while the sequences from PAP2L clade appear to be more diverged and, therefore, were named “PAP2-like”. The PAP2 and PAP2-like sequences are characterized by the presence of three catalytic motifs (C1: KxxxxxxRP, C2: PSGH and C3: SRxxxxxHxxxD) inside the acidPPc domain of PAP2 and predominantly 5–6 transmembrane domains (TMDs). Most of the differences among the clades lie in the C1 and C3 motifs, although their impact on the catalytic activity of the domain remains to be elucidated. Proteins of the PAP2-1 and PAP-2 clades are similar in length (~400 amino acids), while the PAP2L sequences are shorter (~320 amino acids on average). Several PAP2L sequences turned out to be pseudogenes or had incorrectly annotated gene borders.

The distribution of the PAP2 orthologs in Trypanosomatidae is most compatible with the scenario suggesting the presence of the PAP2-1 and PAP2-2 sequences in the trypanosomatid common ancestor. Another copy of PAP2 gene appeared in the ancestor of trypanosomatids after the genus *Trypanosoma* branched off the tree, thus giving rise to PAP2L. The respective genes were duplicated in *Crithidia expoeki* and likely in *Blechomonas ayalai*, while being present as a single copy in the majority of other species (Fig 1). The PAP2L in *L. major* and *L*. *turanica* lack C1 and C2 domains and are probably not functional, suggesting that pseudogenization also played a certain role in the evolution of this protein family. The genome of *L*. *guyanensis* M4147 (this study) encodes two PAP2 and one PAP2L sequences, preserving all conserved catalytic residues in 3 motifs (Fig 1). The targeted PAP2 of *L*. *guyanensis* belongs to the PAP2L clade.

**Fig 2:**
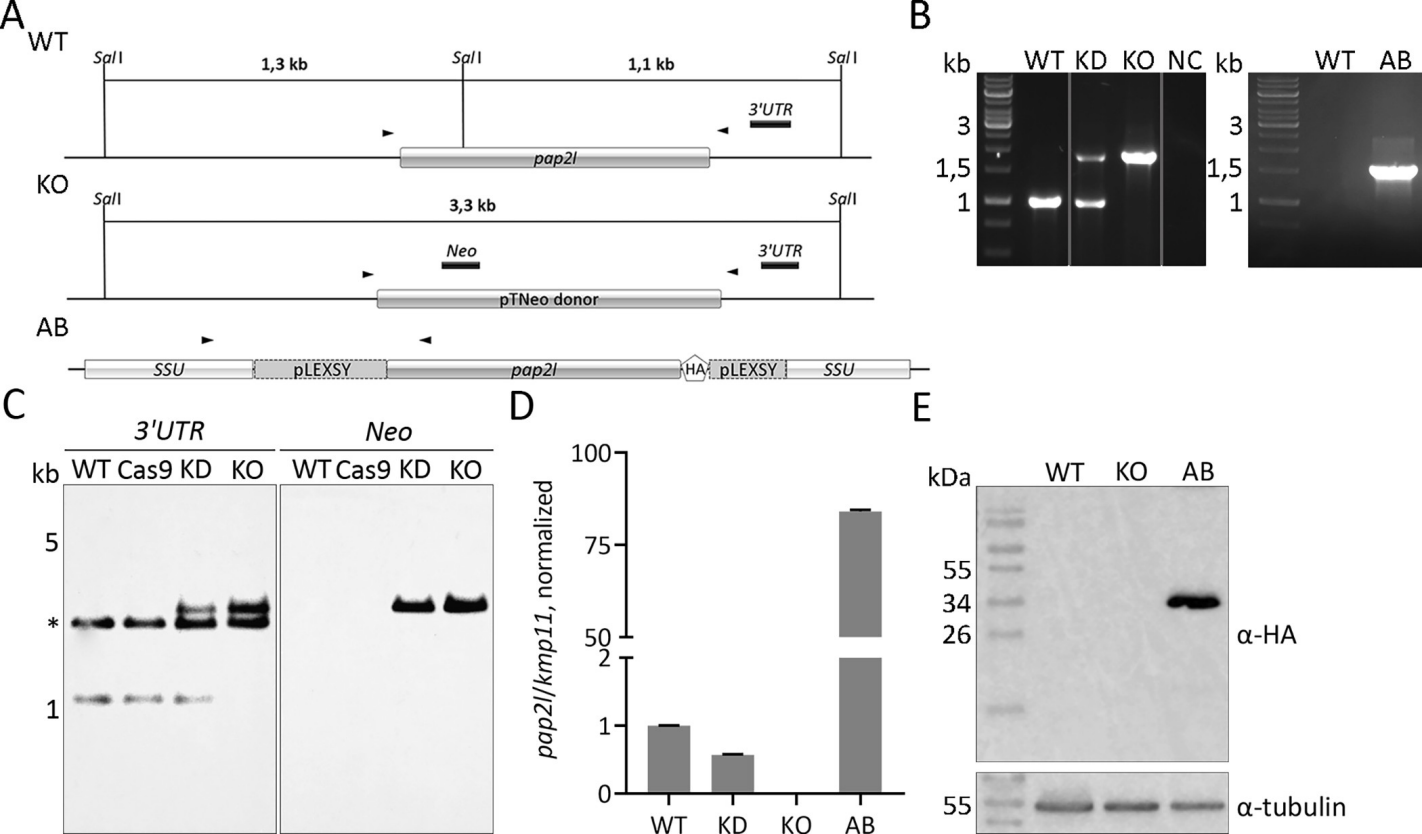
Genetic ablation of *pap2l* using CRISPR-Cas9 approach in *L*. *guyanensis*. (A) Schematic representation of the wild type (WT), recombined (KO), and reintroduced (AB) *pap2l* alleles. Expected sizes of DNA fragments after *Sal*I digestion, positions of the annealed probes for Southern blotting and primers for diagnostic PCR (arrowheads) are indicated. (B) Diagnostic PCR for the WT, KD and KO (left) and AB (right) *pap2l* alleles in *L*. *guyanensis*. NC, negative control. (C) Southern blotting analysis of the *Sal*I-digested total genomic DNA of the WT, Cas9/T7, KD, and KO *L*. *guyanensis*. Membranes were probed with *3′UTR* and *Neo* probes. A non-specific band in anti-*3′UTR* analysis is marked by the asterisk. (D) Quantitative RT-PCR analysis of *pap2l* expression in the WT, KD, KO, and AB *L*. *guyanensis*. Data were normalized to the expression of *kmp11* and presented as means and standard deviations of three independent biological replicates. (E) Western blotting confirmation of the *Lg*PAP2L-HA expression in the AB line. Probing with anti-tubulin antibody served as a loading control.

The *pap2l* gene was deleted using two gRNAs that annealed to the 5′ and 3′ portions of the ORF and replacing it with Neomycin resistance gene (Neo) (Fig 2A and S1 Table). As a result, we obtained *L*. *guyanensis pap2l*^+/-^ knock-down (KD) and *pap2l*^-/-^ knock-out (KO) cell lines. Successful integration and replacement of either one or two alleles was confirmed by PCR (Fig 2B, left, expected fragment sizes for the wild type and knock-out alleles are 993 and 1,826 bp, respectively), Southern blotting with *3′UTR* and *Neo* probes (Fig 2C), and RT-qPCR (Fig 2D). Only a line with complete KO was used in all subsequent experiments. Next, we have established the add-back (AB) cell line by integrating HA-tagged *Lg*PAP2L ORF into 18S rRNA locus of *L*. *guyanensis pap2l*^-/-^. A successful integration and expression of the reintroduced gene was confirmed by PCR (Fig 2B, right), RT-qPCR (Fig 2D), and Western blotting with anti-HA antibodies (Fig 2E). We noticed that *pap2l* expression in the AB cells was much higher than that in the WT parasites, but this is not surprising considering the nature of the locus the added-back gene was expressed from. Other PAP-related loci were not affected by these genetic manipulations.

### *In vitro* analyses: morphometry, cell division, viral load, and lipid content

**Fig 3:**
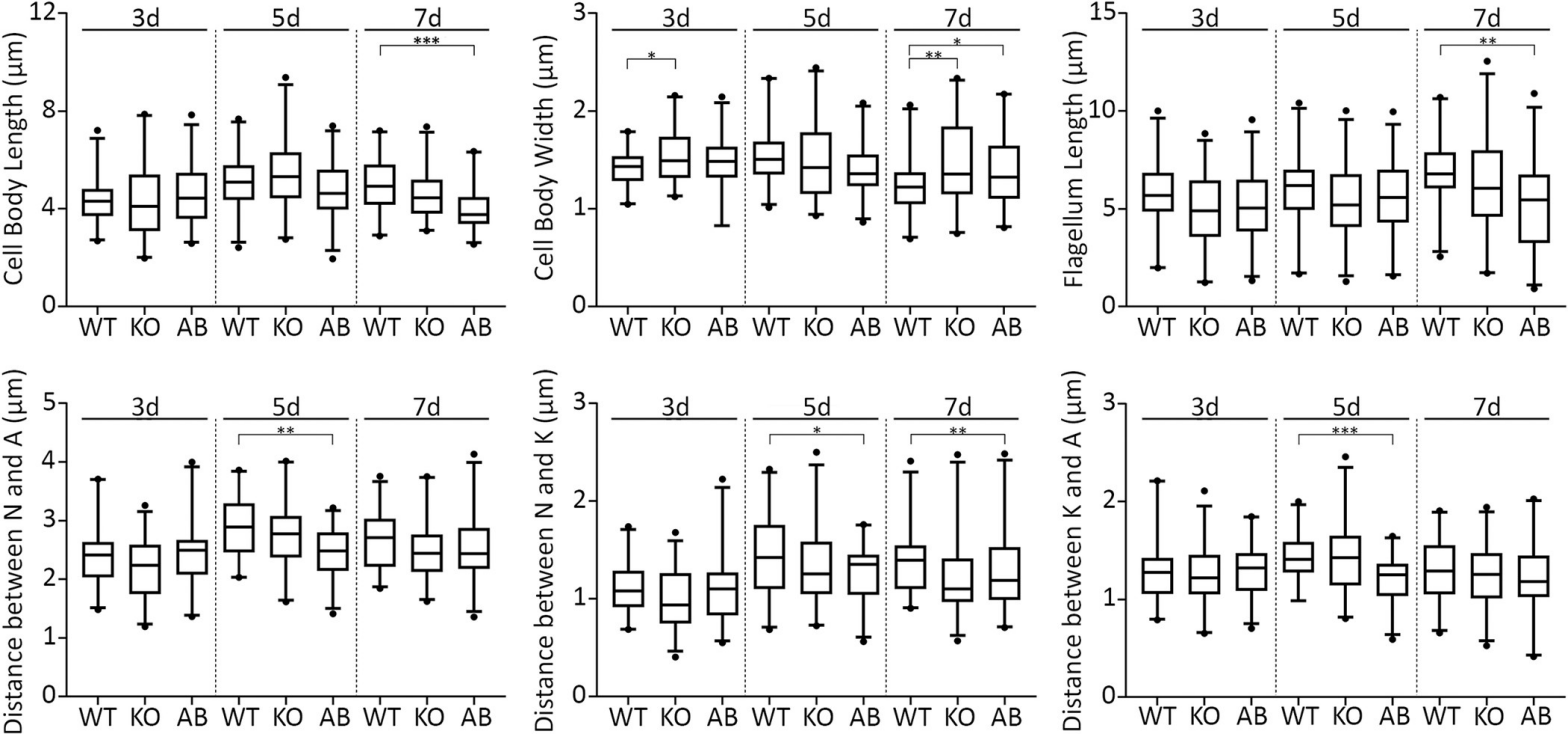
Morphometric analysis of *L*. *guyanensis* with ablated *Lg*PAP2L *in vitro* for days 3, 5, and 7 of cultivation. A, anterior end of the cell; K, kinetoplast; N, nucleus. Boxes and error bars indicate the median along with the upper and lower quartiles, and 97.5 percentiles, respectively. Asterisks show significant differences (*–*P* ≤ 0.05; **–*P* ≤ 0.01; ***–*P* ≤ 0.001), no other differences were statistically significant.

**Fig 4:**
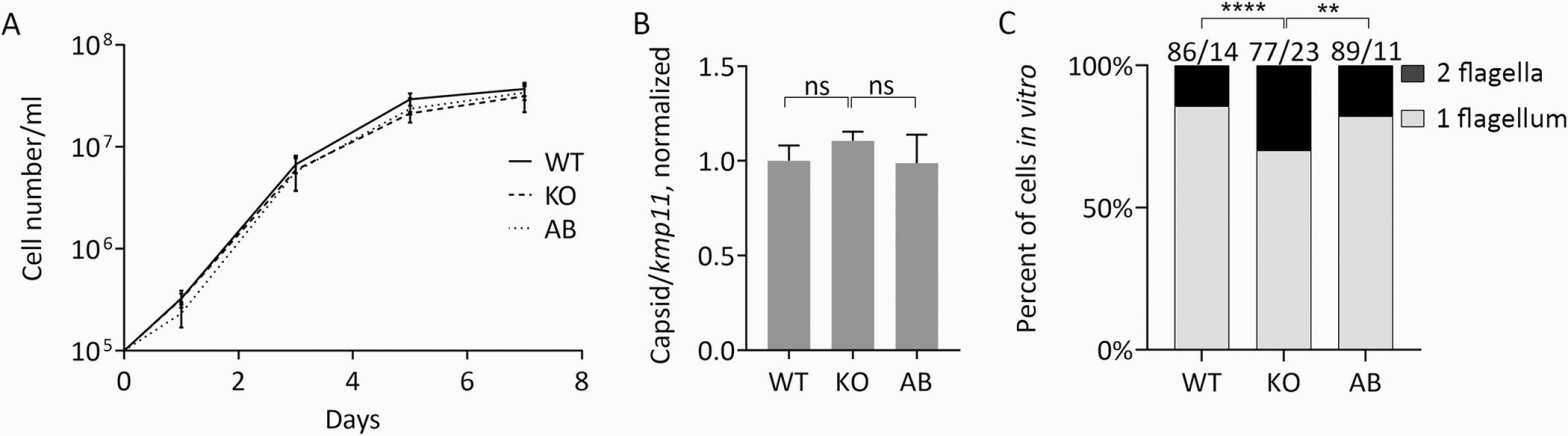
Growth curve, analysis of viral load, and bi-flagellated state of *L*. *guyanensis* with ablated *Lg*PAP2L *in vitro*. A) Growth curves of the WT, KO, and AB *L*. *guyanensis*. (B) RT-qPCR analysis of the LRV1 capsid RNA level in the WT, KO, and AB *L*. *guyanensis* after 5 days of cultivation. Data are presented as normalized fold expression over *kmp11* and reported as the mean and standard deviation of three replicates. Two-tailed Student’s t-test was used for statistical analysis; ns–not significant. (C) The proportion of mono- and bi-flagellated cells in the WT, KO, and AB *L*. *guyanensis* after 5 days of cultivation. Asterisks indicate significant differences (**–*P* ≤ 0.01; ****–*P* ≤ 0.0001).

We first measured the standard morphological traits–cell body length and width, length of the flagellum, and position of the nucleus and kinetoplast–for the WT, KO, and AB *L*. *guyanensis* cells in dynamics on days 3 (~6 × 10^6^ cells/ml), 5 (~2.6 × 10^7^ cells/ml), and 7 (~3.4 × 10^7^ cells/ml) and documented no significant differences (Fig 3). Cell division rate and viral load were also similar between all the investigated cell lines (Fig 4A and 4B). Notably, the proportion of bi-flagellated cells was significantly higher in *L*. *guyanensis* cells lacking *Lg*PAP2L and this phenotype was reverted back to the WT level in the AB parasites (Fig 4C). More detailed analysis (S4 Fig) indicated that KO cells may get halted at the M and post-M phases of their cell cycle (higher proportion of 2K2N2F and 2K1N2F at day 3, and 1K1N2F cells at days 5 and 7).

Prompted by the fact that analyzed protein belongs to the PAP2 superfamily, we decided to investigate the effect its ablation has on the levels of different lipids. The two-dimensional TLC analysis of phospholipids and neutral lipids showed a significant accumulation of phosphatidic acid in the KO cells (5.7 ± 2.8%) compared to that in the WT (1.6 ± 0.3%) or AB (2.6 ± 0.3%) *L*. *guyanensis*. The differences in the levels of other studied lipids (phospholipids: PC, PI, PS, PE, and CL; neutral lipids: ERGO, DAG, LAN, and TAG) were not statistically significant (Table 1).

**Table 1:** Relative levels of phospholipids and neutral lipids in the WT, KO, and AB *L*. *guyanensis*.

	WT	KO	AB
PC	34.2 ± 6.4	32.4 ± 8	34.9 ± 9.1
PE	29.2 ± 3.9	26.6 ± 2.5	25.3 ± 3.6
CL	6.3 ± 4.6	10.5 ± 3.3	8.0 ± 3.2
PS	14.7 ± 10	15.1 ± 6.3	12.8 ± 9.6
PI	10 ± 2	10.3 ± 4.1	13.6 ± 3.7
PA*	1.6 ± 0.3	5.7 ± 2.8	2.6 ± 0.3

ERGO	39.9 ± 6.1	42.6 ± 4.5	40.3 ± 3.4
DAG	2.9 ± 0.7	2.7 ± 1.1	2.5 ± 0.4
LAN	1.8 ± 0.1	1.4 ± 0.4	1.4 ± 0.4
TAG	53.5 ± 5.3	51.6 ± 4.2	53.1 ± 5.4

PC–phosphatidylcholine; PE–phosphatidylethanolamine; CL–cardiolipin; PS–phosphatidylserine; PI–phosphatidylinositol; PA—phosphatidic acid; ERGO–ergosterol; DAG–diacylglycerols; LAN–lanosterol; TAG–triacylglycerols. The asterisk denotes statistical significance *P* ≤ 0.05.

### Development in sand flies

**Fig 5:**
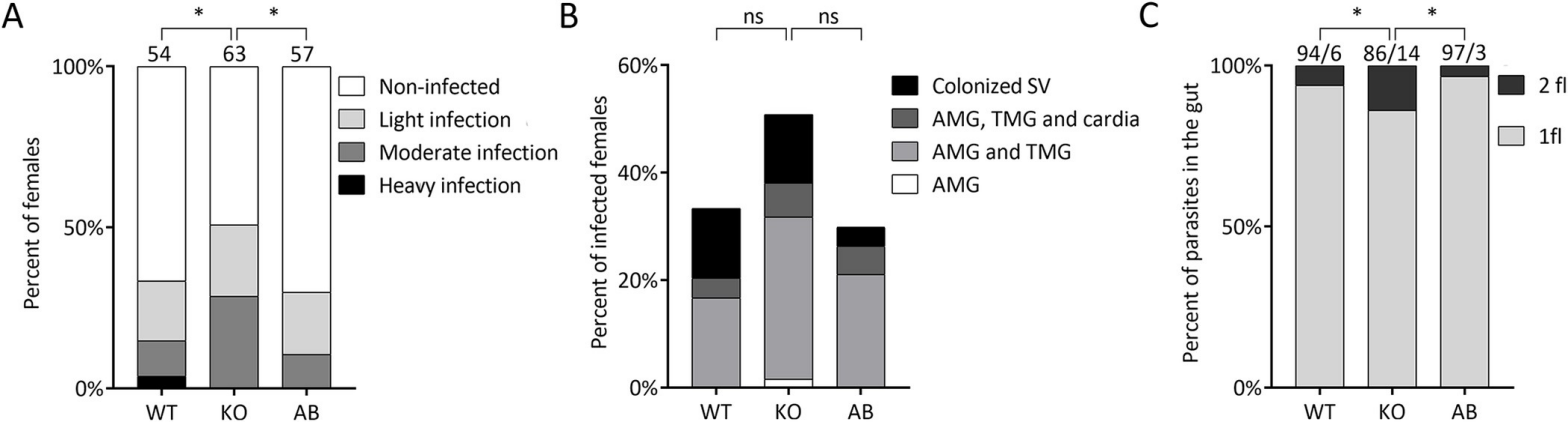
*Leishmania guyanensis* infection in *Lutzomyia longipalpis*. Intensity and localization of infection are presented in panels (A) and (B), respectively. Numbers of dissected females are indicated above the bars in A). AMG, abdominal midgut; TMG, thoracic midgut; SV, stomodeal valve. (C) Percent of mono- and bi-flagellated *L*. *guyanensis* cells 7- and 9-days p.i. in *Lutzomyia longipalpis* gut. *–*P* ≤ 0.05; ns–not significant.

Experimental infections showed that the KO and AB lines were able to survive defecation and develop mature infections in *Lutzomyia longipalpis* comparably to that of the WT parasites. Surprisingly, by day 9 p.i., the infection rate was significantly higher in the KO line (51%) than in both the WT and the AB line (33% and 30%, respectively; *P* = 0.040, d.f. (degrees of freedom) = 2, χ^2^ = 6.442). Intensities of infections also differed significantly (*P* = 0.024, d.f. = 6, χ^2^ = 14.510): moderate or light infections prevailed in all 3 groups, but heavy infections were found only in some females infected with the WT flagellates (Fig 5A). On the other hand, localization of infections did not differ significantly among the groups (*P* = 0.233, d.f. = 8, χ^2^ = 10.481) and mature infections characterized by colonization of the stomodaeal valve were found in females infected with all cell lines (Fig 5B). The *in vitro* observations were confirmed, as there was a higher proportion of bi-flagellated *L*. *guyanensis* cells on d. 9 p.i. in the KO line, compared to both the WT and AB parasites (Fig 5C).

## Discussion

We have obtained a highly contiguous and virtually complete assembly of the LRV1-positive *Leishmania* (*V*.) *guyanensis* genome, totaling ~30.47 Mb and encoding 8,273 protein-coding genes. Among these genes, 6,222 have clear homologues in *V*. *ingenoplastis*. This is not the case for *L*. *guyanensis* PAP2 family protein, which was also identified as one of the *L*. *major* proteins prominently missing from *V*. *ingenoplastis* [34]. We decided to functionally study PAP2L protein and for that it was necessary to establish the CRISPR-Cas9 system. This was achieved, turning this *Leishmania* species into a new model parasite.

The role of PAP2 family proteins was studied mainly in multicellular organisms and bacteria [68,69], while the data for protists is restricted to just a few studies, which highlight the importance of these proteins in the maintenance of PA homeostasis and cell cycle progression [47,48]. Along with diacylglycerol kinases, they regulate phosphatidic acid levels in *T*. *cruzi*. These findings are further corroborated by our study demonstrating that trypanosomatids usually possess several PAP2 family proteins forming 3 main clades on the phylogenetic tree (Fig 1). Our results suggest that all catalytic residues critical for phosphate phosphatase function are conserved in the *L*. *guyanensis* PAP2 sequences including PAP2L, and that at least one of the substrates of the latter enzyme is PA, as judged by the changes in the level of this metabolite in the WT, KO, and AB cell lines. Although the substrate(s) for the other two *L*. *guyanensis* PAP2 homologues remain to be elucidated, it is clear that the expression of the respective genes does not fully compensate for the absence of the *pap2l*-encoded protein leading to the cell cycle defects. Even though the *Lg*PAP2L sequences, closely related to the *L*. *guyanensis* homologue, are absent in several trypanosomatids (S1 Table), the fact that the *L*. *guyanensis pap2l*^-/-^ phenotype is reminiscent of the bi-flagellated *V*. *ingenoplastis* is striking. As a consequence, one is tempted to speculate that (a mutation leading to) the loss of *pap2l* function likely resulted in the cell cycle defect(s) in the latter species. However, since this loss of function might have been outweighed by the ability to resist the midgut peristaltic flow within the fly host, it became fixed and triggered the remarkable morphological speciation, exemplified by extant bi-flagellar *Vickermania* spp. [35]. Moreover, *L*. *guyanensis* with ablated *pap2l* showed higher infection rate in sand flies compared to the WT cells. The most parsimonious explanation of this fact lies in the higher number of more motile bi-flagellated cells, which can better attach to the wall of the digestive tract. It is well-established that the anchoring of *Leishmania* spp. in the insect gut is one of the crucial factors allowing its survival in the vector [70,71]. For future studies, it would be interesting to analyze the effect of *pap2l* ablation in *Leishmania* intracellular amastigotes in mouse or human macrophage infections.

Previous observations suggested that PAP2 or PAP2L are unlikely to be involved in the glycolipid biosynthesis due to its localization in the plasma membrane [72]. Instead, both proteins have been implicated in the conversion of the external bioactive lipids and cellular signaling [73]. In this work, we have confirmed that the relative abundance of cellular phospholipids and neutral lipids was not affected in the *Lg*PAP2L KO, while the PA level has significantly increased in the correspondingly depleted cells. Initially, we have hypothesized that the *Lg*PAP2L deficiency will lead to a reduction in the level of DAGs, but this was not the case. Firstly, the PAP2 and PAP2L enzymes are not specific for PA and can dephosphorylate many other lipid phosphate substrates [41]. Thus, the PA pool is affected just partially when *Lg*PAP2L is ablated. Secondly, several pathways can contribute to the stable level of the DAG pool, for instance *via* hydrolysis of PL and TAG or transfer of the acyl group onto monoacylglycerol [36]. This situation is reminiscent of that in the apicomplexan parasites, *Toxoplasma gondii* and *Plasmodium falciparum*, where PAP2 plays a regulatory role in maintaining the phosphatidic acid homeostasis. Dysregulation of this homeostasis *via* PAP2 inhibition or ablation has affected microneme secretion in *T*. *gondi* and proliferation in both parasites [47,74].

While numerous highly advanced methods for dissecting a handful of model species of the family Trypanosomatidae have been developed [75], they are not always optimized for working with non-model flagellates, many of which are of great medical importance. *Leishmania guyanensis* is one of the few *Leishmania* spp. that frequently carries a symbiotic RNA virus, which aggravates experimental murine leishmaniasis [19] and represents a risk factor for the development of mucocutaneous leishmaniasis [76]. In this respect, there was a need to turn this pathogen into a convenient model amenable to quick and easy genetic manipulations. In this study, we transposed the well-established CRISPR/Cas9 strategy [29] into a *L*. *guyanensis* LRV1-positive strain and confirmed that it did not affect either the fitness of the host culture or the virus. We envision that such a system will allow deeper studies into the mechanisms of interaction between the protist and the virus at the level of molecular factors involved in viral maintenance, which, in turn, may facilitate finding better therapeutic strategies in the treatments of leishmaniases caused by *L*. *guyanensis* and other virus-carrying leishmaniae. In addition, it may promote exploration of the novel virulence factors of *Leishmania* spp. and evolutionary studies of these fascinating medically-relevant parasites and their viruses.

## Materials and methods

### Ethics statement

Animals used for blood-feeding of sand fly colonies were maintained and handled in the animal facility of Charles University in Prague, in accordance with institutional guidelines and Czech legislation (Act of the Czech National Assembly on the Protection of Animals Against Cruelty No. 246/1992, latest amendment No. 359/2012), which complies with all relevant European Union guidelines for experimental animals. All experiments were approved by the Committee on the Ethics of Laboratory Experiments of the Charles University in Prague and were performed under the permit MSMT-8604/2019-6 and Certificate of Competency (Registration Number: CZ 02552).

### Cultivation, total DNA and RNA isolation and next-generation sequencing

*Leishmania guyanensis* MHOM/BR/75/M4147 (the “gold standard” in *L*. *guyanensis* research with available isogenic virus-free lines [19,77,78]) was purchased from the American Type Culture Collection (Manassas, USA) and cultivated in M199 medium (Millipore Sigma, Burlington, USA) supplemented with 2 μg/ml Biopterin (VWR, Radnor, USA), 2 μg/ml Hemin (BioTech, Prague, Czech Republic), 25 mM HEPES, 100 units/ml of penicillin, 100 μg/ml of streptomycin (all from VWR), and 10% Fetal Bovine Serum (BioTech) at 23°C as described previously [79]. The species identity was confirmed by diagnostic PCR and sequencing as in [80]. Total DNA and RNA was isolated and sequenced as described previously [81] at Macrogen Europe (Amsterdam, the Netherlands) and Institute of Applied Biotechnologies (Prague, Czech Republic). The TruSeq DNA PCR-free protocol was used for genomic DNA library; it was sequenced on NovaSeq 6000 resulting in ~55M 150 bp paired-end reads. Three TruSeq stranded mRNA libraries were sequenced on the same platform resulting in ~125M reads.

### Genome assembly and annotation, variant calling

Prior to the assembly, genomic reads were trimmed using Fastp [82] with the following settings: ‘detect_adapter_for_pe’, ‘trim_poly_g’, ‘overrepresentation_analysis’, ‘cut_front’, ‘cut_front_window_size 1’, ‘cut_front_mean_quality 3’, ‘cut_tail’, ‘cut_tail_window_size 1’, ‘cut_tail_mean_quality 3’, ‘average_qual 30’, and ‘length_required 75’. The same settings were applied to transcriptomic reads, except for the ‘cut_front_mean_quality’, ‘cut_tail_mean_quality’, and ‘average_qual’ set to the defaults, ‘length_required’ set to 50 and ‘trim_poly_x’ option ‘on’. The read quality and adapter content were checked before and after the trimming with FastQC v. 0.11.9 [83]. Approximately 50M trimmed genomic reads were assembled *de novo* using SPAdes genome assembler v. 3.13.0 [84] with default settings and automatic k-mer selection (k-mers of lengths 21, 33, 55, and 77 nt were tested). The resulting 7,774 scaffolds (~31 Mbp) were checked for potential contamination with BlobTools v. 1.1.1 [85]. The scaffolds shorter than 500 nucleotides or showing high-quality BLAST hits (sequence identity at the nucleotide level > 95% and coverage > 85%) to non-euglenozoan sequences in NCBI nt database were discarded. Using these criteria, 6,529 scaffolds (889,910 bp) were removed as short sequences and 18 (14,490 bp) were identified as contamination (S1 Fig). Mitochondrial DNA was identified by submitting all scaffolds with GC content below 40% to BLASTn against the NCBI nt database [86], resulting in identification of 100 scaffolds (~95 Kbp) corresponding to kinetoplast DNA (kDNA). After removal of putative contaminants and kDNA sequences, the assembly was scaffolded with MeDuSa v. 1.6 [87] using ‘-w2’ and 10 cleaning rounds, resulting in 39 scaffolds. The genome assemblies of *Leishmania braziliensis* MHOM/BR/1975/M2904 (TriTrypDB, release 55 [88]), *L*. *panamensis* MHOM/PA/1994/PSC-1 (TriTrypDB), and *L*. *guyanensis* isolate 204–365 (NCBI, BioProject PRJNA484340) were used as references. The newly scaffolded assembly was gap-filled with the GapCloser v. 1.12 module from SOAPdenovo2 [89] with the default settings and average insert size of 430 (estimated using Picard CollectInsertSizeMetrics v. 2.25.5 of GenomeAnalysisTK v. 4.2 [90]http://www.broadinstitute.github.io/picard), and maximum read length of 151. The assembly was corrected using RagTag v. 2.1.0 [91] with the default settings and genome of *L*. *panamensis* MHOM/PA/94/PSC-1 (TriTrypDB, [92]) selected as a reference based on its contiguity and BUSCO v.5 [93] completeness. Trimmed paired-end reads were used for the assembly validation. The corrected assembly was ordered and oriented with RagTag using genome of *L*. *braziliensis* MHOM/BR/75/M2904_2019 (TriTrypDB, [94]). The final scaffolded assembly was once again gap filled using the trimmed reads as described above. The quality of the intermediate and the final genome assemblies was verified using SQUAT [95] and QUAST v. 5.0.2 [96].

The repeats in the genome assembly were soft-masked with RepeatMasker v. 4.1.2-p1 [97] using the sensitive slow search, HMMER as search engine, and the option ‘-species’ set to ‘euglenozoa’. Genome annotation using transcriptomic evidence was performed on Companion web server [98] with the default options and *L*. *braziliensis* as the most closely related available reference genome assembly. The genome completeness and annotation quality were assessed with BUSCO with the ‘eukaryota_odb10’ as a reference database. The genome read mapping was performed using Bowtie2 v. 2.2.5 [99] using “-end-to-end” and “-very-sensitive” settings. The transcriptome reads were mapped with BBMap v. 38.18 [100] with the following settings: slow k = 12, requirecorrectstrand = t, ambiguous = random, secondary = f, local = t.

After mapping, the read duplicates were removed and the reads were locally realigned using the MarkDuplicates and IndelRealigner tools of GenomeAnalysisTK v. 4.2 with the default settings, except for REMOVE_DUPLICATES = true. Variant calling was performed using Platypus v. 0.8.1 [101] with the default settings.

The raw reads and assembled genome sequences were deposited to the NCBI database under BioProject accession number PRJNA808737 (BioSample SAMN26112928, accession number JAKSZV000000000).

### Synteny analysis

Synteny analysis was performed using SyMAP v. 5.0.5 [102] with the following settings: minimum size of sequence to load = 500 bp; minimum number of anchors required to define a synteny block = 7; synteny blocks merged in case of overlaps; only the larger block kept if two synteny blocks overlapped on a chromosome. The kDNA sequences were removed from all genomes prior to this analysis and all ‘gene’ features were considered.

### PAP2 phylogenetic analysis

The orthologs of *L*. *guyanensis* PAP2L sequence were searched using BLASTp with an *e*-value threshold of 10^−10^ in the dataset of 50 trypanosomatid genome-derived proteomes (S1 Table). In cases when no ortholog could be identified among annotated proteins, the tBLASTn searches with the same settings were performed using unannotated genomes as a database. This resulted in identification of 155 putative PAP2 and PAP2-like sequences with a minimal sequence identity of 28% and minimal coverage of 20%. These sequences were aligned using the L-INS-i algorithm from MAFFT v. 7.487 [103] with the default settings, and the resulting alignment was trimmed using trimAl v. 1.4.1 [104] with ‘-gappyout’ option and used for the phylogenetic tree inferences.

The maximum-likelihood tree was built using IQ-Tree v. 1.6.12 [105] with the default settings except adding protein mixture models “-madd C10,C20,C30,C40,C50,C60,LG4M,LG4X” to the model selection process [106,107]. The LG + F + R5 model was selected as best fitting according to the Bayesian information criterion [108]. Branch supports were estimated with 1,000 standard bootstrap replicates. The tree was rooted at midpoint and visualized in FigTree v. 1.4.4 [109]. The TMDs were predicted using TMHMM Server v. 2.0 [110] with default settings. Sequence logos were generated using WebLogo [111].

Homologs between *L*. *guyanensis* and *V*. *ingenoplastis* were verified through BLASTP searches using *e-*value cut-off of 0.05. Only hits with e-value ≤ 10^−10^ or with bit-score ≥ 50 were considered and confirmed using a reciprocal BLASTP analysis with a cut-off value of 0.05. This retrieved 6,222 *L*. *guyanensis* proteins with homologs in *V*. *ingenoplastis*, and 1,749 *L*. *guyanensis* proteins with no clear homologs.

### Metabolic predictions

The *L*. *guyanensis* protein sequences were used as query in “all against all” BLASTP searches (with a cut-off value of 10^−20^) against the proteomes of selected trypanosomatids available from the TriTrypDB. One hundred thirty-two proteins present in *L*. *guyanensis* were absent in the *L*. *major* genome at a cut-off of 10^−50^, while 434 *L*. *major* proteins were not detected in *L*. *guyanensis* (excluding hypothetical proteins). Peroxisomal targeting sequences (C-terminal PTS1 and N-terminal PTS2) were identified by searching the *L*. *guyanensis* proteome as described previously [112]. ClustalW [113] was used for the alignment of sequences and the calculation of pairwise distances.

### Reconstruction of LRV1 sequence from total transcriptomic reads

Trimmed reads of *Leishmania guyanensis* WT replicates were independently assembled *de novo* with Trinity v. 2.8.4 [114,115] with ‘—min_contig_length 2000’ and ‘—no_normalize_reads’ options. Reads were mapped onto the LRV1- LgyM4147 sequence (NCBI accession number KX808487) with Bowtie v. 2 2.3.5.1 [99] with default settings.

### Establishment of CRISPR-Cas9 system and genetic manipulations of *L*. *guyanensis*

The previously optimized CRISPR-Cas9 strategy for *Leishmania mexicana* [29] was applied to *L*. *guyanensis*. In brief, cells were transfected with 3 μg of the pTB007 plasmid [66], the resulting populations and clones were selected in liquid and solid complete M199 medium supplemented with 50 μg/ml Hygromycin (Roche Life Science, Penzberg, Germany). Expression of the Cas9-FLAG was confirmed by reverse transcription-quantitative PCR (RT-qPCR) using the gene encoding Kinetoplastid membrane protein 11 (*kmp11*) for normalization (hereafter, all the primer sequences are listed in S2 Table) [116] and by Western blotting as described previously [117]. One clone with the highest expression of Cas9 was chosen for subsequent experiments.

The guide (g)RNAs and donor DNAs for ablation of the gene of interest (encoding *Lg*PAP2L protein) were amplified as described earlier [29]. Populations were selected in liquid M199 medium supplemented with an additional 50 μg/ml Hygromycin and 100 μg/ml Neomycin (InvivoGen, San Diego, USA). Deletion of gene of interest was confirmed by PCR, RT-qPCR and Southern blotting as in [81]. The gene add-back was done via integration of the HA_3_-tagged target gene in the 18S rRNA locus as described previously [118]. The expression of the add-back gene was confirmed by RT-qPCR and Western blotting with anti-HA antibodies.

### Analysis of viral load

A Direct-zol RNA kit (Zymo Research, Irvine, USA) was used for the extraction of total RNA from 5 × 10^7^ mid log phase *L*. *guyanensis* cells. The cDNA was synthesized with random hexamer primers using the Transcription First Strand cDNA Synthesis Kit (Roche Life Science) following the manufacturer’s recommendations. The presence of LRV1 in *L*. *guyanensis* was confirmed by RT-qPCR with primers designed for LRV1 capsid (S2 Table) as previously described [119]. The experiments were done in triplicates using *kmp11* as a housekeeping gene for normalization [81].

In addition, cells were harvested by centrifugation at 1,000 × g for 10 min at 4°C, washed with 1 × phosphate buffered saline (PBS, Millipore Sigma), settled on slides, fixed with 4% paraformaldehyde in PBS for 20 min at room temperature and then washed with PBS for 30 min. The immunofluorescent microscopy for dsRNA was performed as described previously [120,121] after cell staining with the mouse monoclonal anti-dsRNA J2 antibody (Scicons, Szirák, Hungary), followed by the goat anti-mouse IgG–Alexa Fluor-488 antibody (Thermo Fisher Scientific, Waltham, USA) combined with 4′,6-diamidino-2-phenylindole (DAPI, Millipore Sigma). Images were captured on the Olympus microscope BX-53 (Olympus, Tokyo, Japan) equipped with the Olympus DP73 digital camera. They were further processed by Olympus cellSens v.1.6 software and pseudo-colored in ImageJ v.1.51n [122].

### Analyses of morphology and growth kinetics *in vitro*

Cell morphology (cell body length and width, nucleus and kinetoplast position, nucleus size, and flagellum length) was analyzed for 150 cells as in [123,124]. Proportion of mono- and bi-flagellated cells was calculated for 300 cells. Growth kinetics *in vitro* was analyzed for 7 days from the starting density of 1 × 10^5^ cells/ml. Cell numbers were counted in 3 biological replicates using a hemocytometer every 48 hrs. as described previously [120]. Cell cycle was analyzed as in [125–127]

### Sand fly infection

The sand fly colony of *Lutzomyia longipalpis* (originating from Brazil) was maintained in the insectary of the Department of Parasitology, Charles University in Prague, under standardised conditions (26°C, fed on 50% sucrose, with 14 hrs. light/10 hrs. dark photoperiod) as described previously [128]. Promastigotes from a log-phase culture were resuspended in heat-inactivated defibrinated ram blood (LabMediaServis, Jaroměř, Czech Republic) at a concentration of 1 × 10^6^ promastigotes/ml. Sand fly females were infected by feeding through a chick-skin membrane and engorged specimens were maintained in the same conditions as the colony for subsequent dissections. Intensity and localisation of infection on day 9 post-infection (p.i.) were evaluated under the light microscope as described previously [118]; the infections were scored as light (<100 parasites per gut), moderate (100–1,000 parasites per gut), or heavy (>1,000 parasites per gut). Guts with medium or heavy infections were smeared on glass slides, fixed with methanol and stained with Giemsa for subsequent morphological analysis as in [129].

### Analysis of lipids

Total lipids were isolated from 5 × 10^8^ cells by Folch method [130]. Briefly, pelleted cells were homogenized by sonication in total volume of 3 ml chloroform: methanol (2: 1; v/v) in Pyrex borosilicate glass tubes (Thermo Fischer Scientific). Homogenates were incubated for 2 hrs. at 56°C. The organic phase was separated after addition of 500 μl of water. Extracted lipids present in organic phase were dried under nitrogen stream and resuspended in a chloroform: methanol mix (2: 1; v/v).

Diacylglycerols (DAG, products of the PAP2-mediated reaction) were analyzed after neutral lipid separation on silica TLC Kieselgel 60 plates (Millipore, Sigma), which were developed twice using the first mobile phase [petroleum ether: diethyl ether: glacial acetic acid (35: 15: 1; v/v/v)] and second mobile phase [(petroleum ether: diethyl ether (49: 1; v/v)]. Spots were visualized after spraying with 10% CuSO_4_ in 10% phosphoric acid followed by the development with a hot air. Relative lipid content was determined using CAMAG WinCATS software after scanning TCL plates on CAMAG TLC scanner 3 (CAMAG, Muttenz, Switzerland) at 473 nm. Spots were identified by comparing their Rf values with those of standards.

For the detection of phosphatidic acid (PA, a substrate of PAP2), lipids were separated in two dimensions on silica TLC Kieselgel 60 plates as described previously [131]. The mobile phases for the first and second dimensions were chloroform: methanol: ammonium hydroxide: water (66: 27: 3: 0.8; v/v/v/v) and chloroform: methanol: glacial acetic acid: water (32: 4: 5: 1; v/v/v/v), respectively. Spots were visualized and identified as above. All chemicals and lipid standards were from the Millipore Sigma and Avanti Polar Lipids/ Croda International Plc (Birmingham, USA), respectively.

### Statistical analysis

The statistical analysis was performed using GraphPad Prism v. 9 (GraphPad Software, San Diego, USA). The Kolmogorov–Smirnov test was used to determine whether values show normal distribution. For datasets with Gaussian distribution, the ANOVA-Tukey or the two-tailed Student’s t-test were used. For the non-normal datasets, the Mann-Whitney test was applied. Differences in intensity and localization of infection were tested by χ^2^ test in IBM SPSS Statistics v. 27.0.1.0 (IBM, Armonk, USA).

## Supporting information

S1 Fig BlobTools statistics for *Leishmania guyanensis* M4147 genome before (left) and after (right) decontamination. Coverage plots (top) and allocation validation (bottom) are shown.
(TIF)

S2 Fig Synteny analysis of *L*. *guyanensis* M4147 genome. Schematic representation of the two-way synteny among the genome of *L*. *guyanensis* M4147 sequenced in this study and those of *L*. *guyanensis* 204–365 and LgCL085, *L*. *panamensis* PSC-1, and *L*. *braziliensis* M2904_2019 (labeled M2904). Direct and inverted synteny blocks are in red and green, respectively. The kDNA sequences were removed prior to the synteny analysis and only chromosomes carrying regions of synteny in the pairwise comparisons are shown.
(PDF)

S3 Fig Establishment of Cas9/T7-expressing *L*. *guyanensis*. (A) RT-qPCR and (B) anti-FLAG Western blotting confirmation of *Cas9* expression in populations 1–2 and clones 1–5. Wild type *L*. *guyanensis* (WT) was used as negative control. Protein standard sizes in B) are in kDa. (C) Growth curves of WT and Cas9/T7-expressing *L*. *guyanensis* (clone 5). Presented data summarize three independent biological replicates.
(TIF)

S4 Fig Cell cycle analysis. Nucleus (N), kinetoplast (K) and flagellum (F) configuration in *L*. *guyanensis* WT, KO, and AB cultures at days 3, 5, and 7 of cultivation *in vitro*.
(TIF)

S1 Table Dataset of 50 trypanosomatid representatives (155 PAP2-related sequences) used in the maximum-likelihood phylogenetic analysis. The sequences are grouped taxonomically. Sequence length and the number of TMDs are indicated. The correspondence between the clades on the phylogenetic tree (Fig 1) and sequences in the table is shown using colors and clade abbreviations. Catalytic motifs for each PAP2 sequence are shown.
(XLSX)

S2 Table Primers used in this study. (XLSX)

S3 Table Genome assembly statistics. Statistics of the *Leishmania guyanensis* M4147 whole-genome and transcriptome sequencing, along with genome assembly and annotation statistics for *L*. *guyanensis* 204–365, *L*. *guyanensis* LgCL085, *L*. *braziliensis* MHOM/BR/75/M2904_2019, *L*. *panamensis* MHOM/PA/94/PSC-1, and *L*. *major* Friedlin.
(XLSX)

S4 Table Metabolic proteins of *L*. *guyanensis*. (XLSX)

S1 File Predicted metabolism of *L*. *guyanensis*. (DOCX)
