# Supplementary figures and images for: Leishmania guyanensis M4147 as a new LRV1-bearing model parasite: Phosphatidate phosphatase 2-like protein controls cell cycle progression and intracellular lipid content
Source: PLoS Negl Trop Dis. 2022 Jun 24;16(6):e0010510. doi: 10.1371/journal.pntd.0010510 (PMC9232130; doi:10.1371/journal.pntd.0010510)

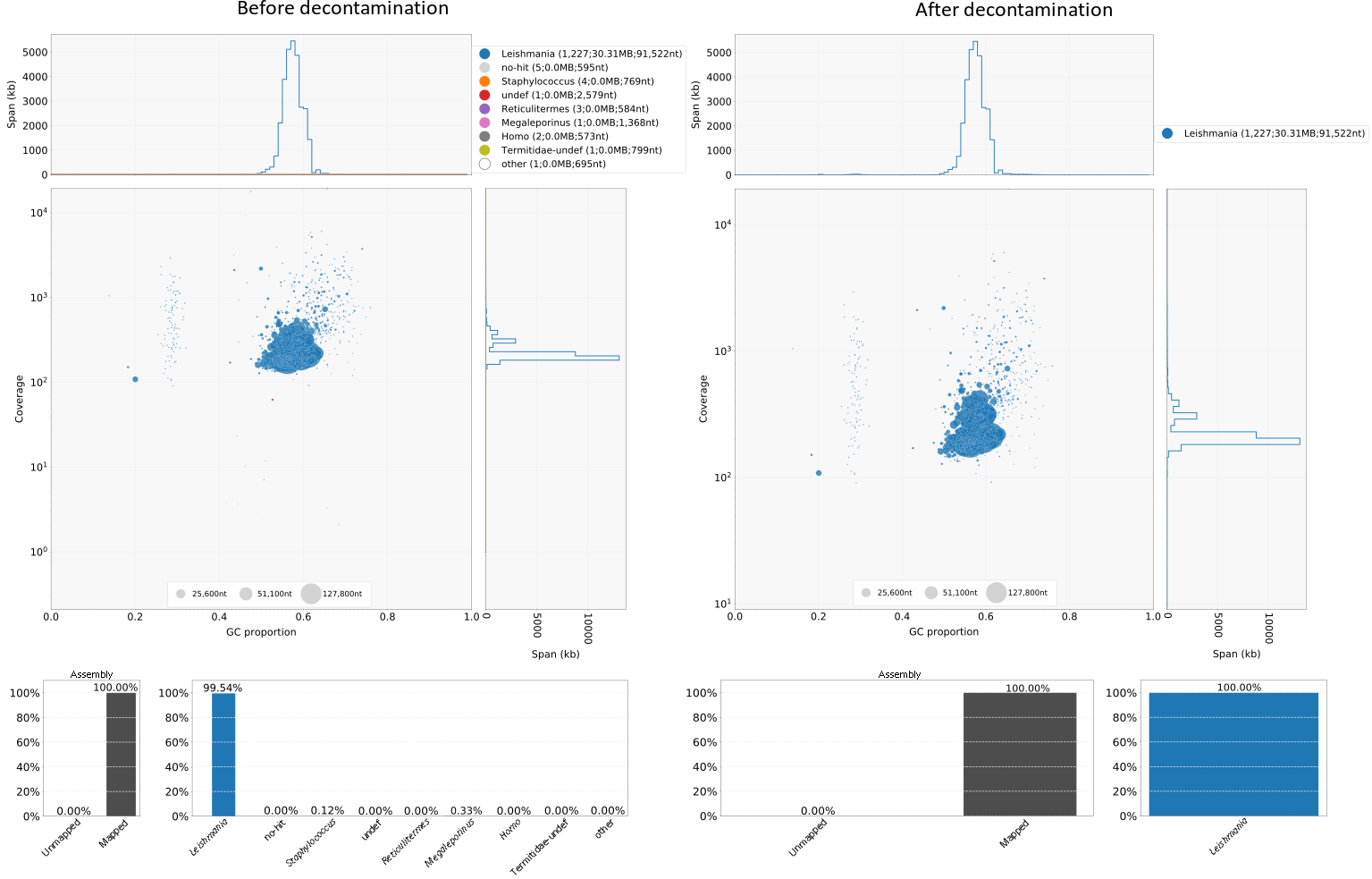

Supplement: S1 Fig — Coverage plots (top) and allocation validation (bottom) are shown. (TIF) [file pntd.0010510.s001.tif]

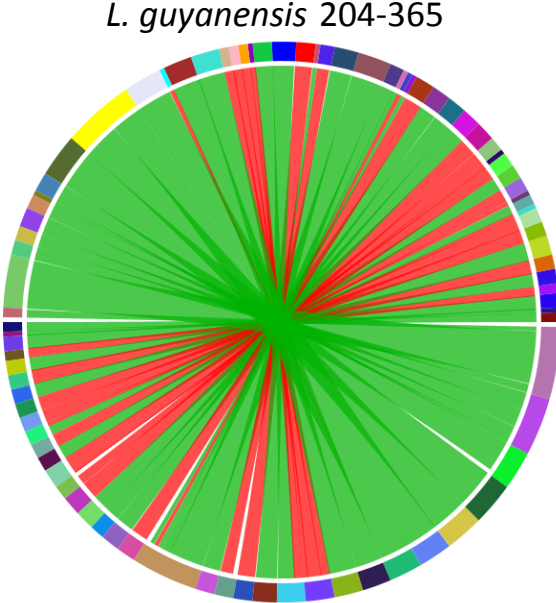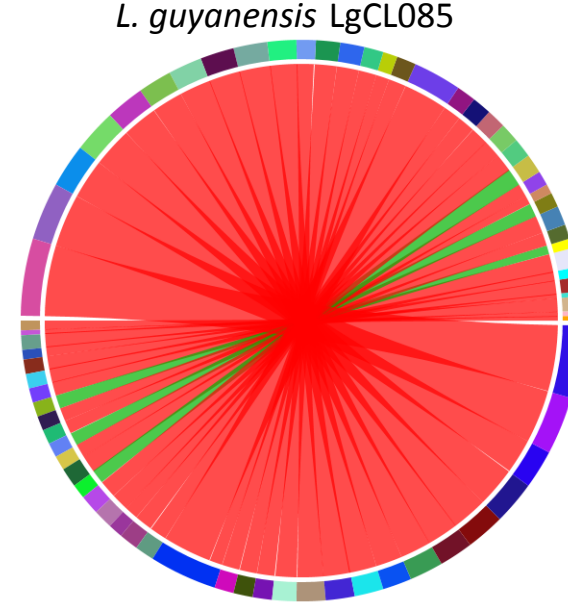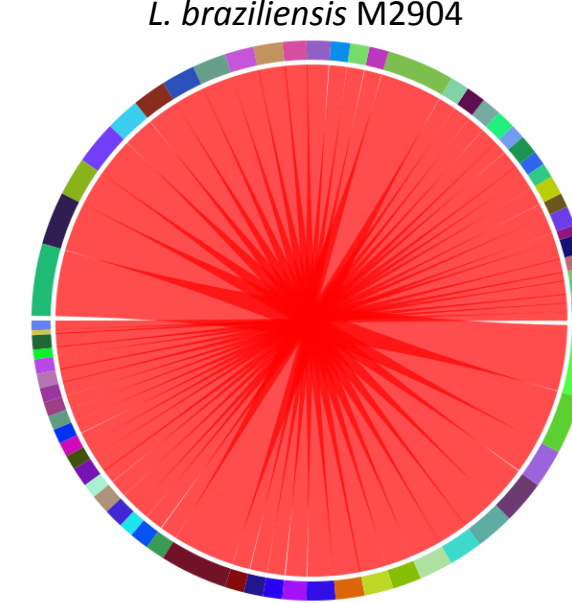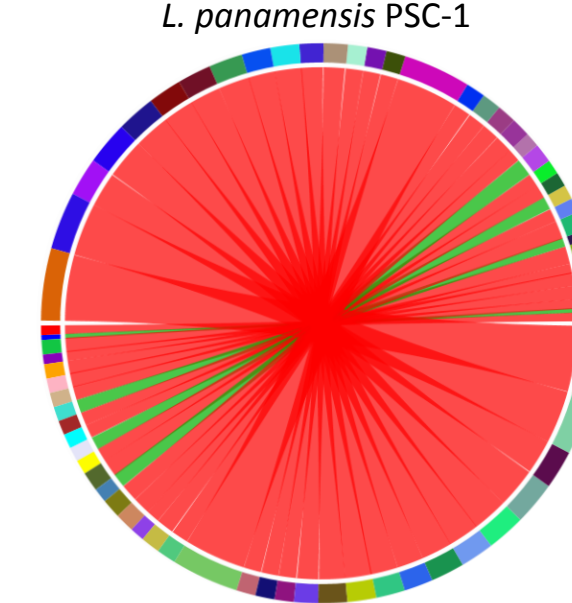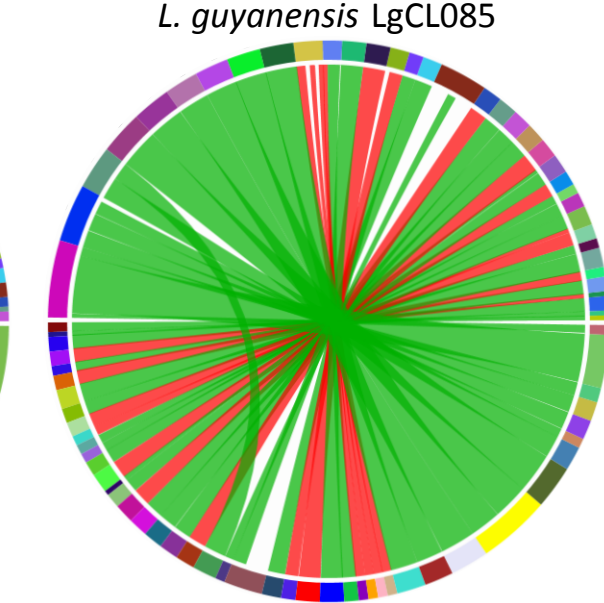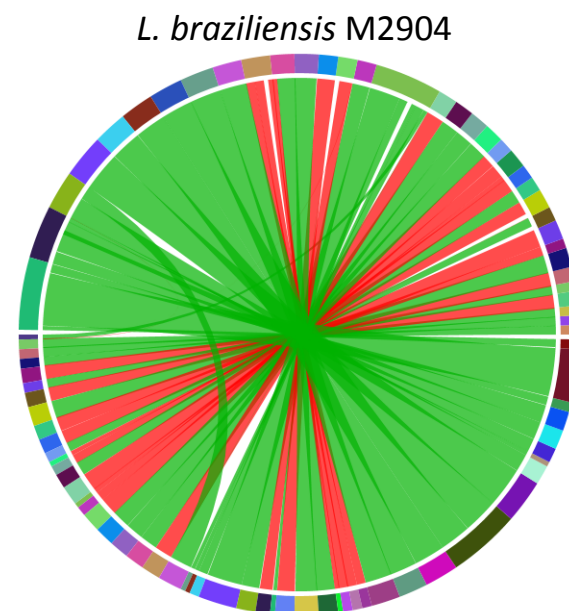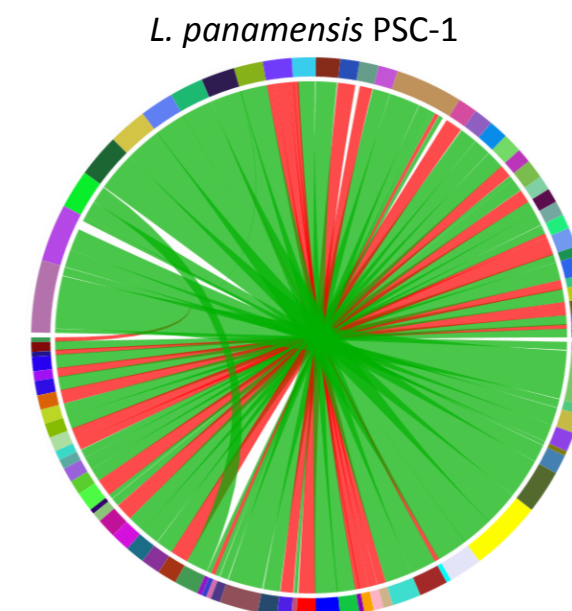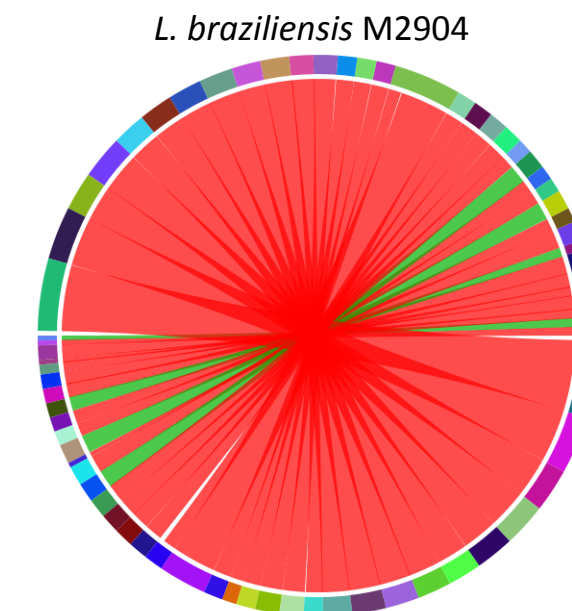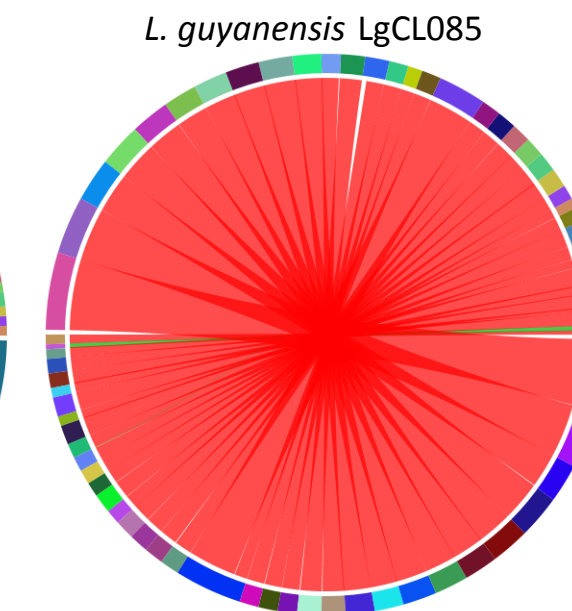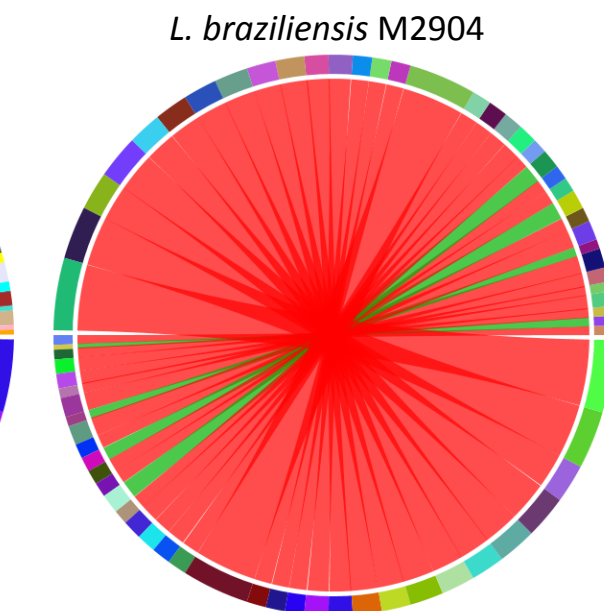

Supplement: S2 Fig — Schematic representation of the two-way synteny among the genome of L. guyanensis M4147 sequenced in this study and those of L. guyanensis 204–365 and LgCL085, L. panamensis PSC-1, and L. braziliensis M2904_2019 (labeled M2904). Direct and inverted synteny blocks are in red and green, respectively. The kDNA sequences were removed prior to the synteny analysis and only chromosomes carrying regions of synteny in the pairwise comparisons are shown. (PDF) [file pntd.0010510.s002.pdf]

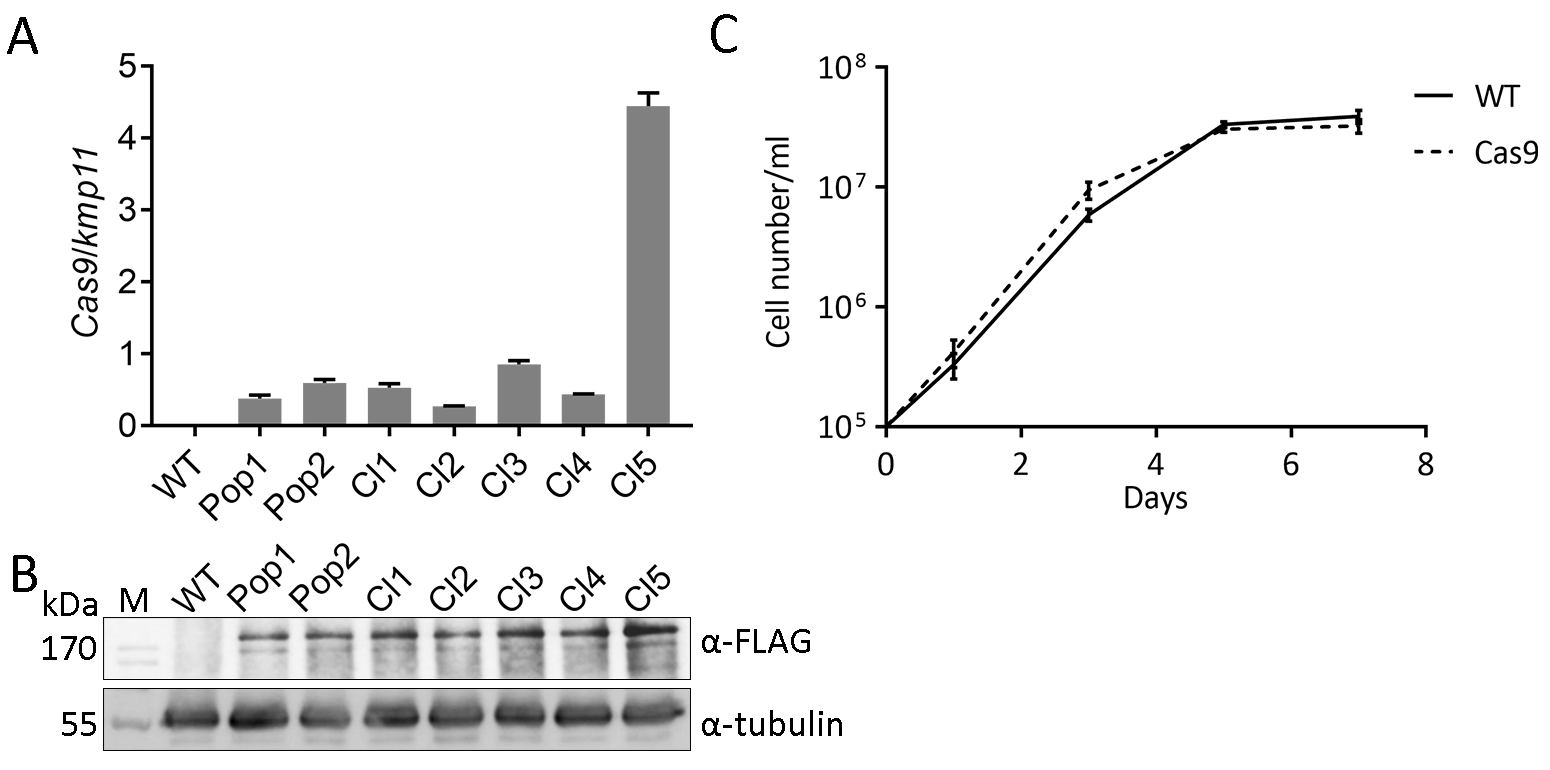

Supplement: S3 Fig — (A) RT-qPCR and (B) anti-FLAG Western blotting confirmation of Cas9 expression in populations 1–2 and clones 1–5. Wild type L. guyanensis (WT) was used as negative control. Protein standard sizes in B) are in kDa. (C) Growth curves of WT and Cas9/T7-expressing L. guyanensis (clone 5). Presented data summarize three independent biological replicates. (TIF) [file pntd.0010510.s003.tif]

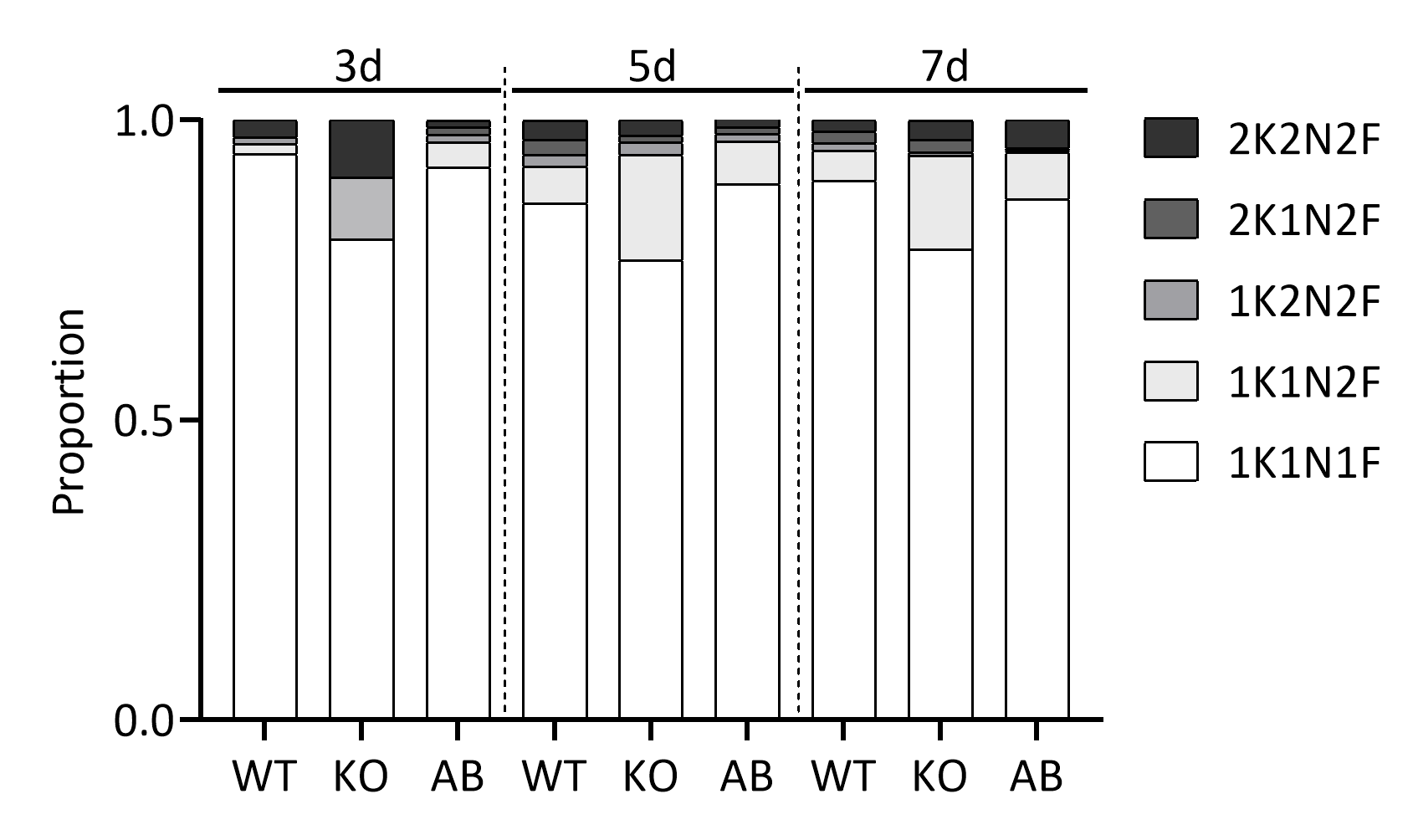

Supplement: S4 Fig — Nucleus (N), kinetoplast (K) and flagellum (F) configuration in L. guyanensis WT, KO, and AB cultures at days 3, 5, and 7 of cultivation in vitro. (TIF) [file pntd.0010510.s004.tif]
