# Supplementary material for: Leishmania guyanensis M4147 as a new LRV1-bearing model parasite: Phosphatidate phosphatase 2-like protein controls cell cycle progression and intracellular lipid content
Source: PLoS Negl Trop Dis. 2022 Jun 24;16(6):e0010510. doi: 10.1371/journal.pntd.0010510 (PMC9232130; doi:10.1371/journal.pntd.0010510)
Supplement: S1 File — (DOCX) [file pntd.0010510.s009.docx]

**Predicted metabolism of** ***L. guyanensis***

Since the metabolism of *L. guyanensis* is very similar to that of its model relative *L. major*, we will focus only on selected aspects of it. In *L. guyanensis* (as in other Leishmaniinae), glycosomes contain several glycolytic enzymes [1], along with proteins involved in gluconeogenesis, fatty acid oxidation, pyrimidine biosynthesis, purine salvage and ether-lipid biosynthesis [2], yet conspicuously lack catalase [3]. This conclusion is based on the identification of predicted peroxisomal targeting signals and on the presence of peroxins, a set of proteins instrumental in the biogenesis of these organelles (S4 Table). Due to the presence of acidocalcisomal exopolyphosphatase and pyrophosphatase, *L. guyanensis* is predicted to carry acidocalcisomes (S4 Table). While all trypanosomatids are heme auxotrophs [4], the proteome of Leishmaniinae (including *L. guyanensis*) possesses three enzymes involved in heme biosynthesis, namely coproporphyrinogen III oxidase, protoporphyrinogen oxidase, and ferrochelatase (S4 Table). Likely acquired *via* HGT, the function of these enzymes in *Leishmania* spp. remains unclear [5]. Genes encoding various heme-containing constituents of the mitochondrial respiratory chain, such as subunits of the mitochondrial *bc1* complex, cytochromes *c* and *c1*, as well as several nuclear-encoded subunits of the cytochrome oxidase complex were identified in the genome of *L. guyanensis*, along with genes encoding cytochromes *b5* and *P450*. In trypanosomatids, the TCA cycle cannot oxidize mitochondrial pyruvate to CO_2_ and water, leaving formed by oxidative decarboxylation acetate as the end product. Together with mitochondrial acetate:succinate coA transferase (ASCT):succinyl CoA synthetase (SCoAS), the pyruvate dehydrogenase complex (PDC) converts mitochondrial acetyl-CoA to acetate, forming ATP by substrate-linked phosphorylation [6-8]. The genes for both ASCT and SCoAS are present in the genome of *L. guyanensis* (S4 Table). The NADH formed in the mitochondrial PDC reaction is re-oxidized by molecular oxygen *via* the respiratory chain. We assume that since the *L. guyanensis* genome contains numerous subunits of complex I (S4 Table), the complex is assembled and active, with ATP produced by oxidative phosphorylation. This distinguishes *L. guyanensis* from *L. infantum* and *L. pifanoi*, where complex I is non-functional [9-11]. *L. guyanensis* lacks a functional urea cycle, with only three (adenylosuccinate synthetase, adenylosuccinate lyase, and arginase) of its five enzymes present (S4 Table). The latter enzyme appears to be associated with the glycosomes, as suggested by PTS1 pre-sequence, which agrees with earlier observations in other Leishmaniinae [12,13].

The analyzed flagellate is able to degrade neutral lipids by diacyl- and monoacyl-lipases to free fatty acids and glycerol, which feeds into the glycolytic pathway after phosphorylation to glycerol 3-phosphate by the glycosomal glycerol kinase equipped with PTS1, as its homologue in *T. brucei* [14]. After oxidation to dihydroxyacetone phosphate by mitochondrial glycerol-3-phosphate dehydrogenase, it returns to the glycosomes to be oxidized further. All the enzymes required for the activation and oxidation of fatty acids (*via* the β-oxidation pathway) and their transport to the mitochondrion have been identified in *L. guyanensis* (S4 Table). Moreover, the presence of glycosomal fatty acid oxidase, along with mitochondrial fatty acyl dehydrogenases, indicates the involvement of glycosomes in β -oxidation of fatty acid.

The unique cytosolic fatty acid synthesis described in other trypanosomatids [15] is also present in *L. guyanensis*. After being carboxylated to malonyl-CoA, acetyl-CoA is used in fatty acid elongation reactions, in the absence of canonical cytosolic multi-subunit fatty acid synthase complex type 1, relying on a set of fatty acid elongases [15] (S4 Table). Ether-lipids synthesized in the glycosomes, with the major sterol in the plasma membrane being ergosterol, same as in the related trypanosomatids [16]. *Leishmania guyanensis* can synthesize it as well, as most of the necessary enzymes were detected in its genome (S4 Table).

Finally, same as in other trypanosomatids, *L. guyanensis* lacks the capacity to synthesize purine bases *de novo*, yet it retains the synthesis of pyrimidines [12], with the last two enzymatic steps being catalyzed by a unique bifunctional glycosomal orotidine-5-phosphate decarboxylase/orotate phosphor-ribosyltransferase endowed with a PTS1 (S4 Table).

**References**

1. Opperdoes FR, Borst P (1977) Localization of nine glycolytic enzymes in a microbody-like organelle in *Trypanosoma brucei*: the glycosome. FEBS Lett 80: 360-364.

2. Michels PAM, Gualdrón-López M (2022) Biogenesis and metabolic homeostasis of trypanosomatid glycosomes: new insights and new questions. J Eukaryot Microbiol: e12897.

3. Kraeva N, Horáková E, Kostygov A, Kořený L, Butenko A, Yurchenko V, et al. (2017) Catalase in Leishmaniinae: with me or against me? Infect Genet Evol 50: 121-127.

4. Kořený L, Oborník M, Horáková E, Waller RF, Lukeš J (2022) The convoluted history of haem biosynthesis. Biol Rev Camb Philos Soc 97: 141-162.

5. Opperdoes F, Michels PA (2008) The metabolic repertoire of *Leishmania* and implications for drug discovery. In: Myler P, Fasel N, editors. *Leishmania*: after the genome. Norfolk, UK: Caister Academic Press. pp. 123-158.

6. van Hellemond JJ, Opperdoes FR, Tielens AG (1998) Trypanosomatidae produce acetate via a mitochondrial acetate:succinate CoA transferase. Proc Natl Acad Sci U S A 95: 3036-3041.

7. Millerioux Y, Morand P, Biran M, Mazet M, Moreau P, Wargnies M, et al. (2012) ATP synthesis-coupled and -uncoupled acetate production from acetyl-CoA by mitochondrial acetate:succinate CoA-transferase and acetyl-CoA thioesterase in *Trypanosoma*. J Biol Chem 287: 17186-17197.

8. Martin WF, Tielens AGM, Mentel M (2021) Mitochondria and anaerobic energy metabolism in eukaryotes: biochemistry and evolution. Düsseldorf, Germany: De Gruyter. 252 p.

9. Duarte M, Ferreira C, Khandpur GK, Flohr T, Zimmermann J, Castro H, et al. (2021) *Leishmania* type II dehydrogenase is essential for parasite viability irrespective of the presence of an active complex I. Proc Natl Acad Sci U S A 118: e2103803118.

10. Čermáková P, Maďarová A, Baráth P, Bellová J, Yurchenko V, Horváth A (2021) Differences in mitochondrial NADH dehydrogenase activities in trypanosomatids. Parasitology 148: 1161-1170.

11. Rainey PM, MacKenzie NE (1991) A carbon-13 nuclear magnetic resonance analysis of the products of glucose metabolism in *Leishmania pifanoi* amastigotes and promastigotes. Mol Biochem Parasitol 45: 307-315.

12. Opperdoes FR, Butenko A, Flegontov P, Yurchenko V, Lukeš J (2016) Comparative metabolism of free-living *Bodo saltans* and parasitic trypanosomatids. J Eukaryot Microbiol 63: 657-678.

13. Flegontov P, Butenko A, Firsov S, Kraeva N, Eliáš M, Field MC, et al. (2016) Genome of *Leptomonas pyrrhocoris*: a high-quality reference for monoxenous trypanosomatids and new insights into evolution of *Leishmania*. Sci Rep 6: 23704.

14. Hammond DJ, Aman RA, Wang CC (1985) The role of compartmentation and glycerol kinase in the synthesis of ATP within the glycosome of *Trypanosoma brucei*. J Biol Chem 260: 15646-15654.

15. Lee SH, Stephens JL, Englund PT (2007) A fatty-acid synthesis mechanism specialized for parasitism. Nat Rev Microbiol 5: 287-297.

16. Urbina JA (2009) Ergosterol biosynthesis and drug development for Chagas disease. Mem Inst Oswaldo Cruz 104 311-318.
